# ESM1-mediated DNMT3A suppresses cervical cancer metastasis through epigenetic regulation of ID3 expression

**DOI:** 10.1038/s41420-026-03239-z

**Published:** 2026-08-07

**Authors:** Chen-Lin Yu, Chia-Liang Lin, Hsiang-Lin Lee, Hui-Ling Chiou, Pei-Ni Chen, Shun-Fa Yang, Yi-Hsien Hsieh

**Affiliations:** 1 Department of Medical Research, Hsinchu MacKay Memorial Hospital, Hsinchu City, Taiwan; 2 Institute of Biomedical Sciences, MacKay Medical University, New Taipei City, Taiwan; 3 Department of Biochemistry, School of Medicine, Chung Shan Medical University, Taichung, Taiwan; 4 Clinical Laboratory, Chung Shan Medical University Hospital, Taichung, Taiwan; 5 Deptartment of Surgery, Chung Shan Medical University Hospital, Taichung, Taiwan; 6 School of Medicine, Chung Shan Medical University, Taichung, Taiwan; 7 School of Medical Laboratory and Biotechnology, Chung Shan Medical University, Taichung, Taiwan; 8 Institute of Medicine, Chung Shan Medical University, Taichung, Taiwan; 9 Department of Medical Research, Chung Shan Medical University Hospital, Taichung, Taiwan

**Keywords:** Cervical cancer, RNA sequencing, DNA methylation, Cell invasion, RNAi therapy

## Abstract

Cervical cancer remains the fourth most common malignancy among women worldwide, and patients with advanced-stage disease continue to experience poor clinical outcomes despite the availability of targeted therapies. In this study, we investigated the epigenetic role of endothelial cell-specific molecule 1 (ESM1), a soluble proteoglycan, and established an oncogene whose regulatory mechanisms in cervical cancer remain largely unexplored. The epigenetic mechanisms underlying tumor progression remain incompletely understood. Here, we identify ESM1 as a critical epigenetic regulator of cervical cancer malignancy. Integrative analyses of public datasets and clinical specimens revealed that marked ESM1 overexpression correlated with adverse patient prognosis. Functional loss- and gain-of-function studies have demonstrated that ESM1 is essential for maintaining proliferative, clonogenic, migratory, and invasive phenotypes in cervical cancer cells. Transcriptomic profiling revealed that inhibitor of DNA binding 3 (ID3) is a direct downstream tumor suppressor repressed by ESM1. Mechanistically, ESM1 selectively upregulates DNA methyltransferase 3 A (DNMT3A) to induce promoter hypermethylation and transcriptional silencing of ID3. Pharmacological demethylation reactivates ID3 expression and attenuates metastatic capacity. In vivo xenograft and experimental metastasis models validated that ESM1 depletion significantly impaired tumor growth and lung metastasis while increasing ID3 expression. These findings identify the ESM1/DNMT3A/ID3 axis as a novel epigenetic driver of cervical cancer and a potential therapeutic target.

## Introduction

Globally, cervical cancer (CC) ranks as the fourth most common cancer among women, with an estimated 660,000 new cases and 350,000 deaths in 2022, disproportionately affecting women in low- and middle-income countries [1]. Early-stage CC is often curable if detected and treated promptly [2]. However, the prognosis of patients with advanced-stage disease remains poor despite the use of targeted therapy or immune checkpoint inhibitors [3]. This highlights the urgent need to identify new therapeutic targets, which requires a deeper understanding of the molecular mechanisms underlying CC metastasis. High-risk human papillomavirus (HPV) infection is the primary cause of approximately 99% of cases [4]. Studies have shown that the E6 and E7 oncoproteins directly upregulate DNMT expression. This overexpression leads to the aberrant hypermethylation of CpG islands in the promoter regions of critical tumor suppressor genes [5, 6]. However, the molecular mechanisms linking specific epigenetic regulators to CC progression remain incompletely characterized.

Endothelial cell-specific molecule 1 (ESM1) is a soluble proteoglycan that is involved in tumor growth, tumor metastasis, and unfavorable prognosis across multiple cancer types [7]. Owing to its specificity for endothelial cells, ESM1 is also known as endocan and is considered a marker of endothelial cell-mediated angiogenesis and inflammation [8, 9]. High ESM1 expression is correlated with advanced tumor stage, increased metastasis, and poor overall survival [10–12]. ESM1 exerts oncogenic functions by activating pathways such as the PI3K/Akt, MAPK/ERK, JAK/STAT, and WNT/β-catenin pathways, thereby promoting proliferation, migration, angiogenesis, and therapeutic resistance [13]. In CC, ESM1 has been shown to enhance epithelial–mesenchymal transition (EMT) and angiogenesis through PI3K/Akt and HIF-1α/VEGFα/ERK signaling, respectively [14]. Nevertheless, its role in epigenetic regulation in CC remains unclear. Inhibitor of DNA binding 3 (ID3), a helix–loop–helix transcription factor lacking a DNA-binding domain [15], exhibits context-dependent roles in tumor biology [16]. Although ID3 promotes tumor progression in some cancers, it functions as a tumor suppressor in others by inhibiting EMT and metastasis [17, 18]. The biological role of ID3 in cervical cancer remains unclear.

This study suggests that ESM1 is overexpressed in cervical cancer tissues and is correlated with poor patient survival. ESM1 inversely regulates ID3 expression by promoting DNMT3A-mediated DNA methylation, leading to ID3 silencing and enhanced cancer cell proliferation, colony formation, and migration/invasion. These findings reveal a novel epigenetic ESM1 that targets the ID3 axis and identify DNA methylation as a potential therapeutic target in cervical cancer.

## Results

### ESM1 is highly expressed in cervical cancer and is associated with poor overall survival of cervical cancer patients

**Fig. 1:**
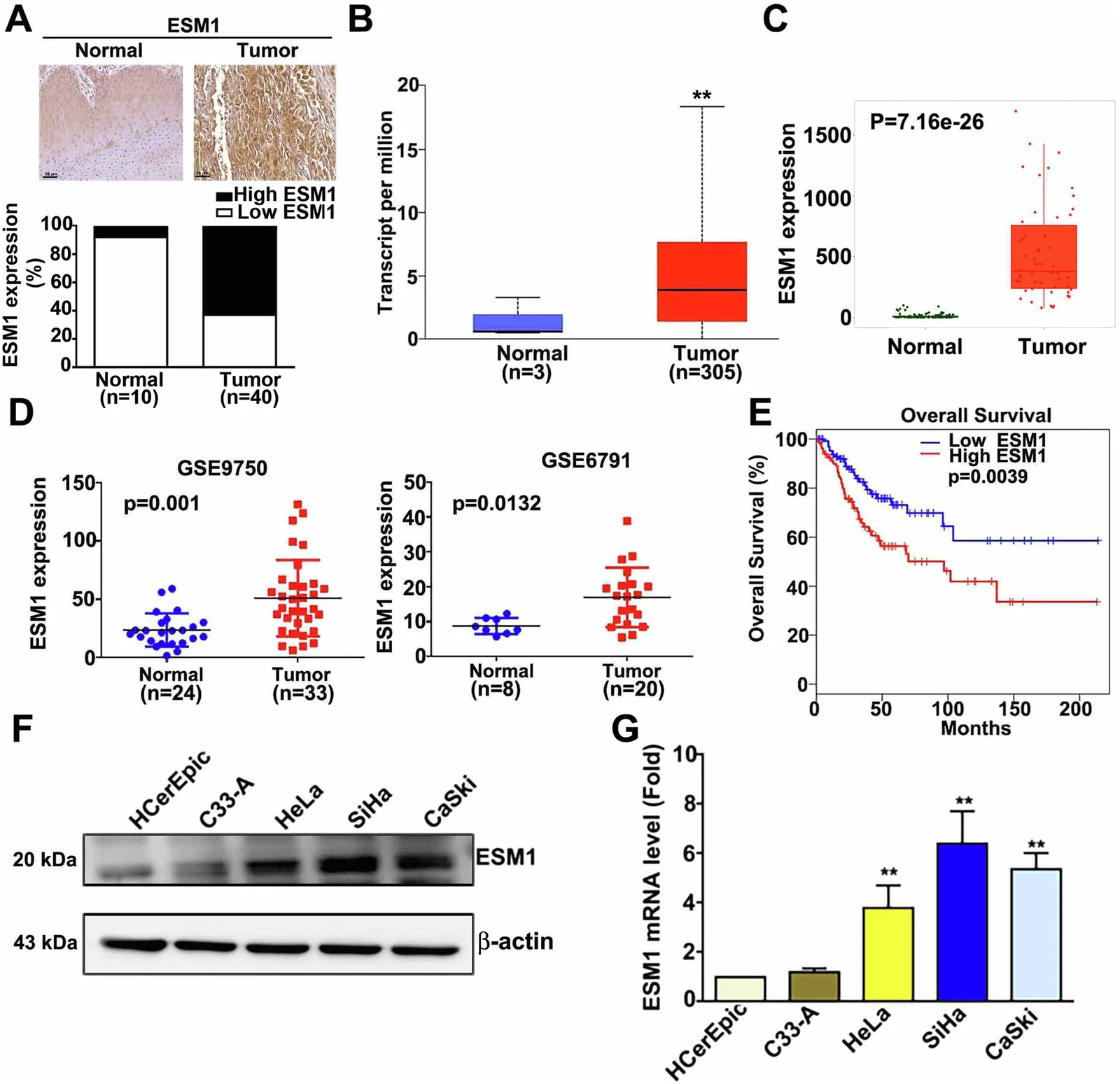
ESM1 is highly expressed in cervical cancer tissue and cervical cancer cell lines and is associated with poor survival in cervical cancer patients. **A** The image above shows a representative immunohistochemistry (IHC) analysis of ESM1 expression in normal and human cervical squamous cell carcinoma tissue. Comparison of the portion of high and low ESM1 expression in normal and cancerous human cervical tissue. Scale bar = 50 μm. **B** Comparison of ESM1 expression between human normal and cancerous cervical tissue from the GEPIA database. **C** Comparison of ESM1 expression between human normal and cancerous cervical tissue from the TCGA database using the TNMplot. **D** Comparison of ESM1 expression between human normal and cancerous cervical tissue from the GEO databases (GSE9750 and GSE6791). **E** Survival analysis of cervical cancer patients with high and low ESM1 expression using the Kaplan-Meier method. **F** The protein and mRNA expression of ESM1 in human cervical epithelial cells (HCerEpic) and four human cervical cancer cell lines were examined by Western blot and RT-qPCR. Data are expressed as the mean ± SD of three independent experiments. Each experiment values are relative to the shLuc group after normalization to β-actin or GAPDH. Fold change relative to the HcerEpic cells. ** *p* < 0.01 compared with the control group or HCerEpic.

To verify the role of ESM1 in human cervical cancer, IHC analysis of cervical tissues was performed with human cervical tissue array. As shown in Fig. 1A, Human cervical squamous cell carcinoma tissues (*N* = 40) exhibited a higher level of ESM1 overexpression than normal cervical tissues (*N* = 10). To further validate the upregulation of ESM1 in cervical cancer, we conducted bioinformatics analyses using multiple public databases. GEPIA2 (Fig. 1B), TNMplot (Fig. 1C), and two GEO databases (GSE9750 and GSE6791) (Fig. 1D) all confirmed our findings in the tissue array. In addition, a Kaplan-Meier analysis using the GEPIA database was performed to elucidate the effect of ESM1 expression on the clinical outcomes of cervical cancer patients, revealing that patients with higher ESM1 expression had lower survival rates (Fig. 1E). Moreover, the difference in the endogenous ESM1 expression in human cervical epithelial cells (HCerEpic) and cervical cancer cell lines, including C33-A (HPV-negative), HeLa (HPV-18 positive), SiHa (HPV16-positive), and CaSki cells (HPV16-positive) was also assessed using immunoblotting and RT-qPCR analysis. We found that the protein and mRNA expression of ESM1 was higher in HeLa, SiHa, and CaSki cells than in the C33-A and in primary human cervical epithelial cells (Fig. 1F, G). Together, these findings clearly indicate that ESM1 overexpression may be crucial for cervical cancer progression.

### Expression of ESM1 is required for the malignant properties of cervical cancer

**Fig. 2:**
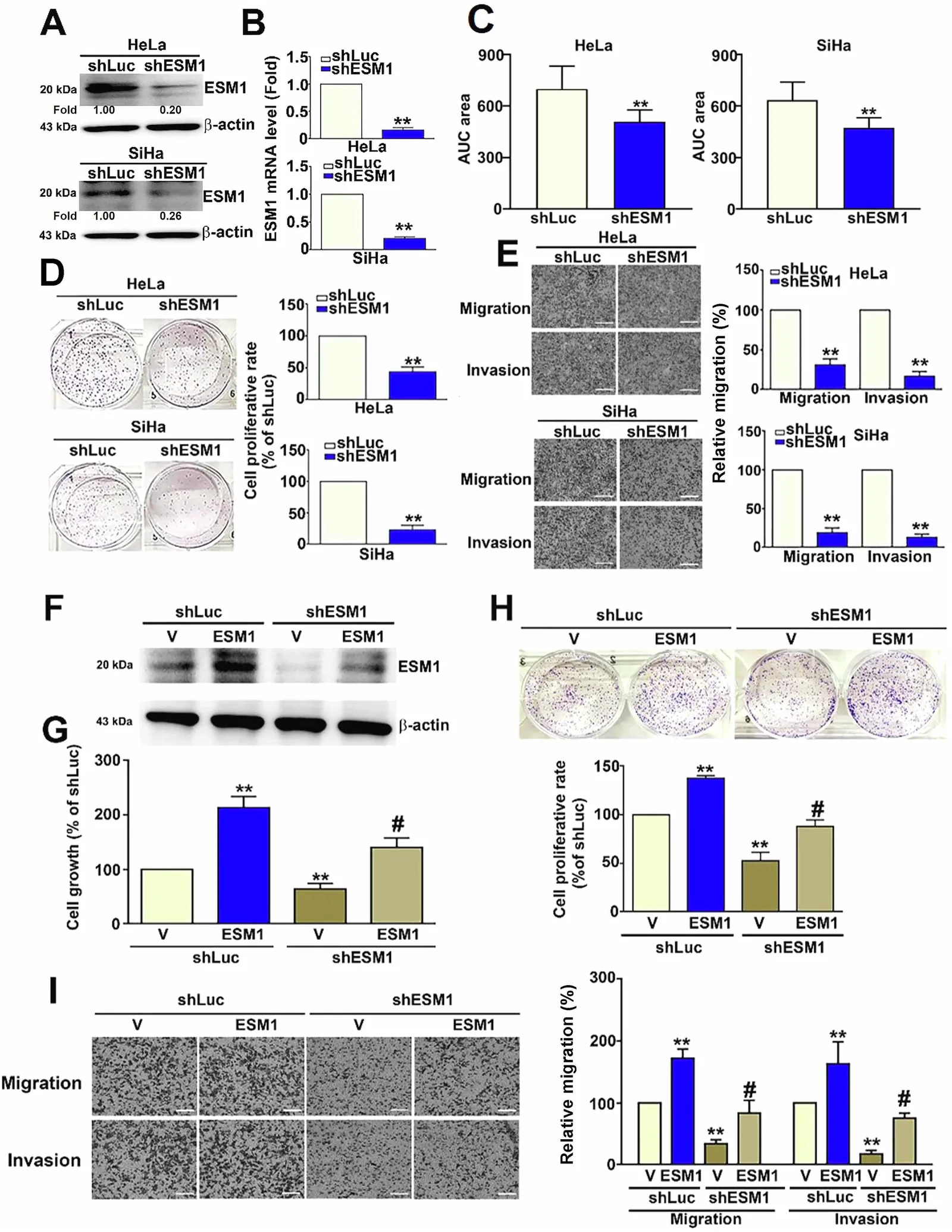
ESM1 regulates the malignant properties of human cervical cancer cells. **A**, **B** The stable knockdown of ESM1 in HeLa and SiHa was confirmed by Western blot and RT-qPCR. **C** The effect of ESM1 knockdown on cell growth was assessed by MTT assay and the AUC was calculated from the proliferation curves using the trapezoidal method. **D** The impact of ESM1 knockdown on cell proliferation was evaluated by colony formation assay. Representative images of surviving colonies and the quantified results were shown. **E** The effects of ESM1 knockdown on migration and invasion were examined using migration and Matrigel invasion assays. Representative images of migrated cells and the quantified results were shown. **F** The reintroduction of ESM1 expression by the ESM1-expressing plasmid was confirmed by Western blot. **G** The effect of reintroducing ESM1 expression on cell growth was assessed by MTT assay. **H** The effect of reintroducing ESM1 expression on cell proliferation was evaluated by colony formation assay. Representative images of surviving colonies and the quantified results were shown. **I** The effects of reintroducing ESM1 expression on migration and invasion were examined using migration and Matrigel invasion assays. Representative images of migrated cells and the quantified results were shown. Original magnification ×630. Data are expressed as the mean ± SD of three independent experiments. Each experiment values are relative to the shLuc group after normalization to β-actin. Fold change relative to percentage (%) relative to the shLuc cells. ** *p* < 0.01 compared with the shLuc control group. # *p* < 0.05 compared with the stable ESM1 knockdown cells transfected with empty vectors.

To further explore the role of ESM1 in cervical cancer progression, we knocked down ESM1 expression using shRNA in HeLa and SiHa cells. As shown in Fig. 2A, B, ESM1 expression was significantly lower in the established ESM1 knockdown HeLa and SiHa stable cells. Next, we evaluate the effect of ESM1 knockdown on growth using MTT by area under the curve (AUC) analysis, colony formation assays, and on metastatic abilities using migration and Matrigel invasion assays. In this study, AUC analysis demonstrated that knockdown of ESM1 treatment significantly suppressed the proliferation kinetics of HeLa (shLuc: AUC 699.30 ± 52.52; shESM1: AUC, 507.35 ± 37.91, *p* < 0.01) and SiHa cells (shLuc: AUC, 701.25 ± 43.49; shESM1: AUC, 471.55 ± 55.83, *p* < 0.01) compared with the shLuc cells (Fig. 2C), colony formation (Fig. 2D), and migration/invasion (Fig. 2E) abilities of cervical cancer cells. To validate the effect of ESM1 knockdown, plasmids containing ESM1 transcripts were transfected into stable ESM1 knockdown cells. After confirming that transfection leads to ESM1 upregulation by immunoblotting, we further examined the effect of restoring ESM1 expression in stable ESM1 knockdown SiHa (Fig. 2F). We found that, by overcoming the ESM1 knockdown, stable ESM1 knockdown SiHa cells have regained their growth (Fig. 2G), colony formation (Fig. 2H), and migration/invasion (Fig. 2I) abilities. These results were consistent with those observed in HeLa cells (Supplementary Fig. 1). Collectively, these results confirmed that ESM1 is essential to the malignant properties of both cervical cancer cells.

### ID3 is inversely correlated with ESM1 cervical cancer

**Fig. 3:**
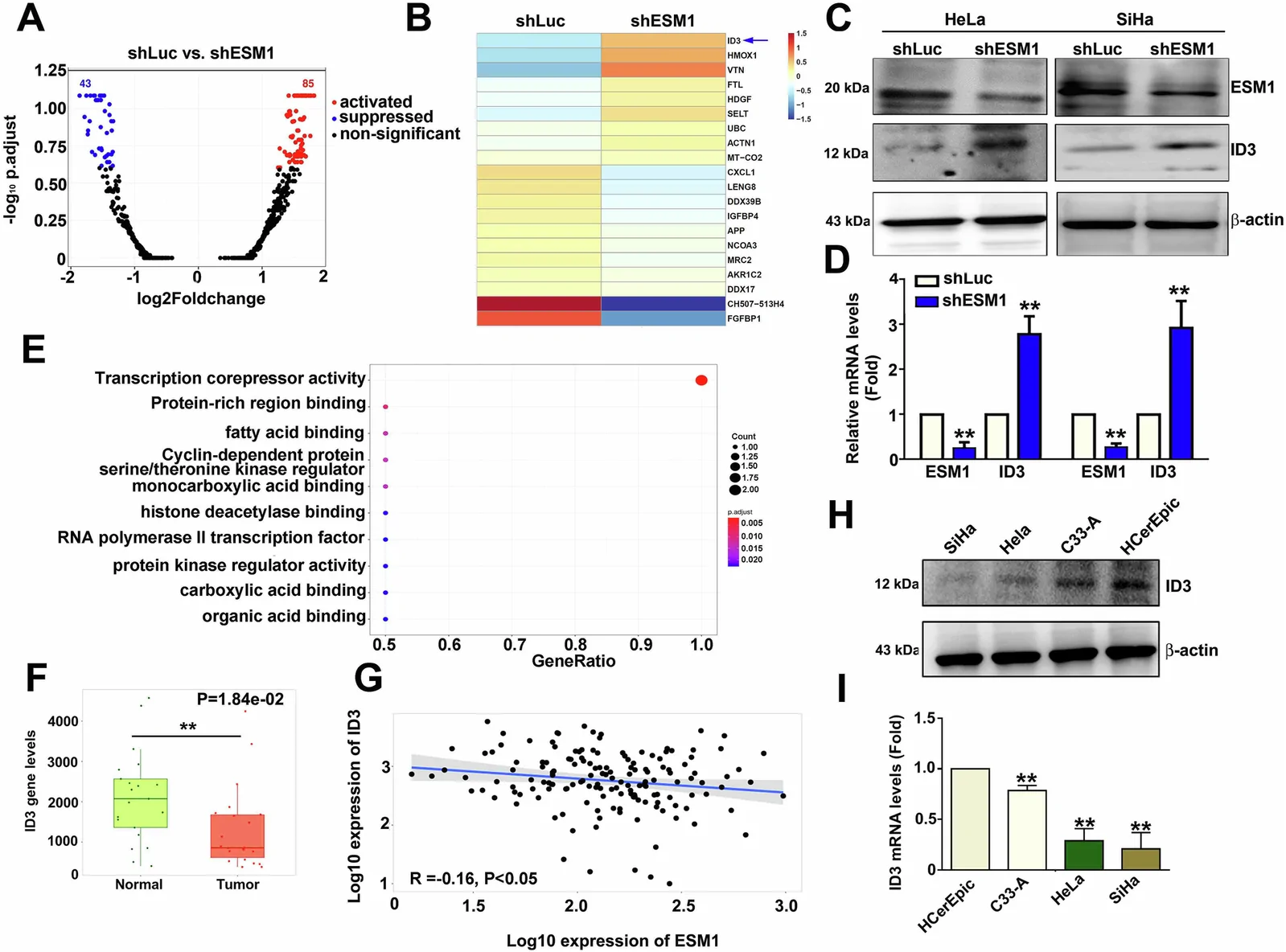
ESM1 knockdown upregulates ID3 expression, and ESM1 and ID3 expression are inversely correlated. **A** The volcano plot of differential expression genes (DEGs) between stable shLuc and shESM1 cervical cancer cells. **B** Heat map of Top 20 DEGs. **C**, **D** The upregulation of ID3 in stable shESM1 cervical cancer cells was verified by Western blot and RT-qPCR. **E** Gene Ontology (GO) enrichment analysis and further described the differentially expressed genes identified from the RNA-sequencing data. **F**, **G** Comparison of ID3 expression between human normal/cancerous cervical tissue and the correlation between ESM1 and ID3 in cervical cancer tissue from the TCGA database using the TNMplot. **H**, **I** The mRNA and protein expression of ID3 in human cervical epithelial cells (HCerEpic) and three human cervical cancer cell lines were examined by RT-qPCR and Western blot. Data expressed as the mean ± SD of three independent experiments. Each experiment values are relative to the shLuc group after normalization to β-actin. Fold change relative to percentage (%) relative to the shLuc cells or HCerEpic. ** *p* < 0.01 compared with the shLuc control group or HCerEpic cells.

To explore the underlying mechanisms of ESM1-mediated cervical cancer progression, RNA sequencing was used to analyze mRNA expression differences in stable ESM1 knockdown cervical cancer cells (Fig. 3A). To identify potential genes involved, the top 20 differentially expressed genes from RNA-seq were ranked by log2 fold change and adjusted *p*-value, and ID3 was the top-ranked gene (Fig. 3B). To validate the RNA-seq finding, immunoblotting and qRT-PCR assay were conducted, and we found that ID3 protein and mRNA expression were indeed higher than in control groups (shLuc) (Fig. 3C, D). Gene Ontology (GO) enrichment analysis further revealed that genes related to ID3 (GO gene set: transcription corepressor activity) were also downregulated in stable ESM1 knockdown cervical cancer cells (Fig. 3E). To further elucidate the connection between ESM1 and ID3, public databases were consulted using TNMplot software. These data showed that ID3 expression was not only lower in cervical cancer tissues than in normal tissues (Fig. 3F) but also negatively correlated with ESM1 in cervical cancer tissues (Fig. 3G). Additionally, to further verify the inverse correlation between ID3 and ESM1 expression, immunoblotting and RT-qPCR were performed to assess the endogenous ID3 levels in HCerEpic and cervical cancer cell lines (C33-A, HeLa, and SiHa). As shown in Fig. 3H, I, ID3 protein and mRNA expressions were higher in low ESM1-expressing cells (HCerEpic and C33-A) and lower in high ESM1-expressing cells (HeLa and SiHa). These data indicate that ID3 expression is inversely correlated with ESM1 expression in cervical cancer.

### ID3 is a bona fide tumor suppressor in cervical cancer

**Fig. 4:**
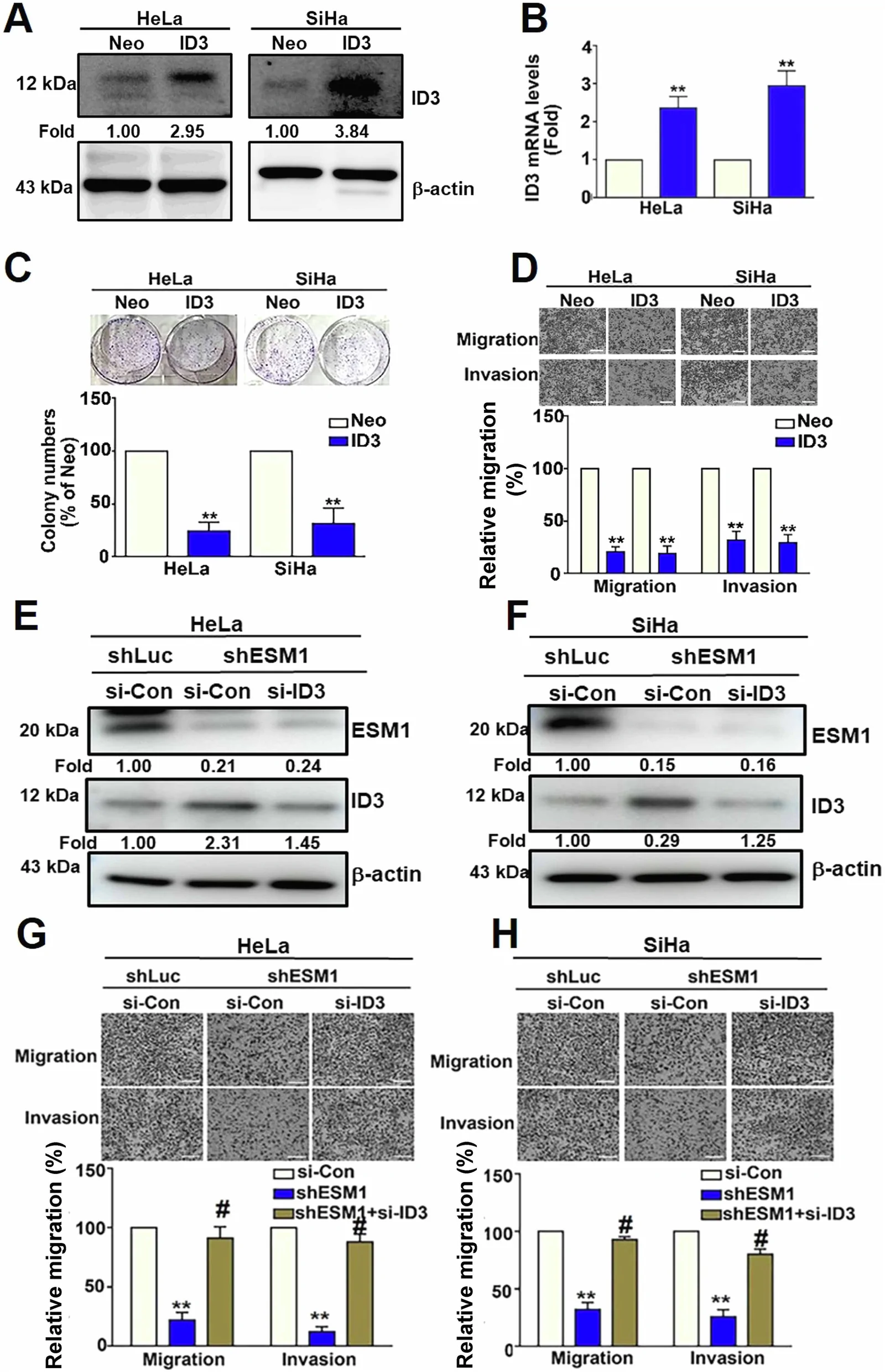
ID3 overexpression suppresses the malignant properties of human cervical cancer cells. **A**, **B** The ectopic expression of ID3 in HeLa and SiHa was confirmed by Western blot and RT-qPCR. **C** The effect of ID3 overexpression on cell proliferation was evaluated by colony formation assay. Representative images of surviving colonies and the quantified results were shown. **D** The effects of ID3 overexpression on migration and invasion were examined using migration and Matrigel invasion assays. Representative images of migrated cells and the quantified results were shown. **E**, **F** The protein expression levels of ESM1 and ID3 were examined by western blot analysis following ID3 silencing in stable ESM1-knockdown HeLa and SiHa cells. **G**, **H** The effects of silencing ID3 in stable ESM1 knockdown HeLa and SiHa cells on migration and invasion were examined using migration and Matrigel invasion assays. Representative images of migrated cells and the quantified results were shown. Data are expressed as the mean ± SD of three independent experiments. Each experiment values are relative to the shLuc group after normalization to β-actin. Fold change relative to percentage (%) relative to the shLuc cells. ** *p* < 0.01 compared with the empty vector or the shLuc control group. # *p* < 0.05 compared with the stable ESM1 knockdown cells transfected with control siRNAs. Original magnification ×630.

Previous studies have shown that the role of ID3 is highly context-dependent [19]. To elucidate the role of ID3 in cervical cancer, ID3-expressing plasmids were transfected into low-ID3-expressing cervical cancer cells (HeLa and SiHa). Immunoblotting and RT-qPCR were used to confirm the ectopic expression of ID3 (Fig. 4A, B). Next, we assess the effects of ID3 overexpression on proliferation and metastatic abilities using colony formation, migration, and Matrigel invasion assays. As shown in Fig. 4C, D, ID3 overexpression significantly impeded the proliferation and migration/invasion of HeLa and SiHa cells. These data suggested that ID3 is a bona fide tumor suppressor in cervical cancer. To further verify the role of ID3 in ESM1-mediated cervical cancer progression, migration assay and Matrigel invasion assay were performed on ID3-targeting siRNA-transfected stable ESM1 knockdown cervical cancer cells. Our results showed that, compared with the control siRNA-transfected stable cells, silencing ID3 significantly decreased the ID3 expression (Fig. 4E, F) and rescued the metastatic abilities (Fig. 4G, H) inhibited by ESM1 downregulation. Taken together, our data demonstrate that ID3 inhibition is pivotal for ESM1-mediated cervical cancer progression.

### ID3 expression is epigenetically regulated in cervical cancer

**Fig. 5:**
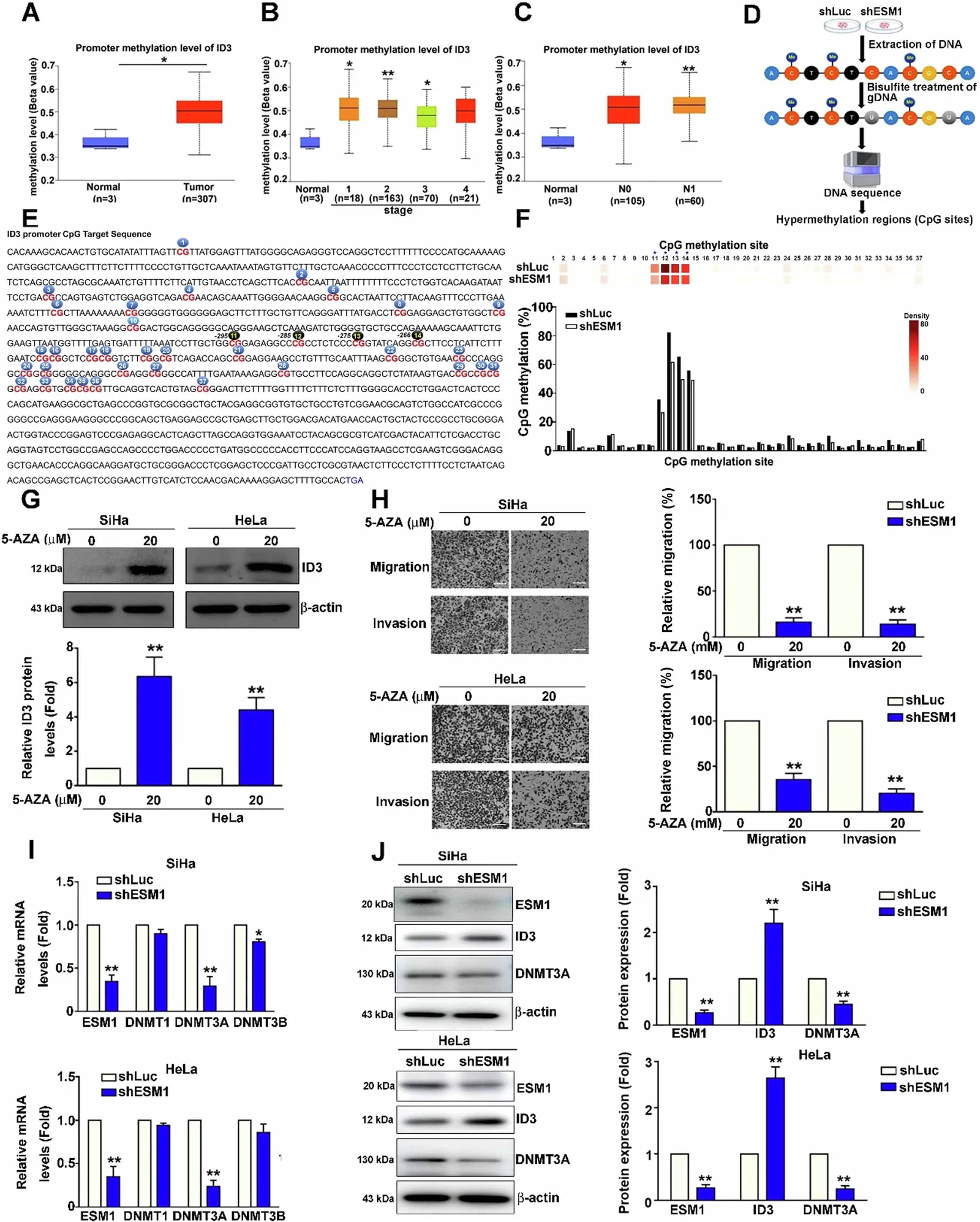
ID3 is epigenetically regulated by ESM1 targeting DNMT3A expression in cervical cancer. Comparison of promoter methylation levels between human normal tissue and **A** total cancerous cervical tissue, **B** cohort of different disease stages and **C** cohort of different metastasis status from the TCGA database using the UALCAN portal. The genomic DNA of stable ESM1 knockdown cervical cancer cells were extracted and treated with bisulfate to solidify the DNA methylation pattern. **D** The schematic diagram illustrates the DNA methylation experimental procedure. **E**, **F** The potential methylation sites in the regulatory region of ID3 promoter were mapped, and the bisulfite-treated genomic DNA was subjected to DNA methylation sequencing to detect the changes in DNA methylation. HeLa and SiHa cells were treated with 20 µM of 5-AZA for 24 h, and the effects of inhibition on (**G**) the expression of ID3 and **H** migration/invasion abilities were examined by Western blot, migration assay, and Matrigel invasion assay. Representative images of migrated cells and the quantified results were shown. **I** The effects of ESM1 knockdown in SiHa and HeLa cells on DNMTs expression (DNMT1, DNMT3A, DNMT3B) were assessed by RT-qPCR. **J** The effects of ESM1 knockdown in cervical cancer cells on the expression of ID3 and DNMT3A were examined by Western blot. Data are expressed as the mean ± SD of three independent experiments. Each experiment values are relative to the shLuc group after normalization to β-actin. Fold change relative to percentage (%) relative to the shLuc cells. * *p* < 0.05, ** *p* < 0.01 compared with the control group.

Epigenetic dysregulation has been shown to play a fundamental role in cervical cancer progression [5]. To determine whether ID3 expression is epigenetically regulated in cervical cancer, we analyzed the TCGA database using UALCAN (The University of ALabama at Birmingham CANcer data analysis). The results indicated that the methylation level of the ID3 promoter region is significantly higher in cervical cancer tissues than in normal tissues (Fig. 5A). Moreover, higher ID3 promoter methylation is not only correlated with higher stage but also with lymph node metastasis (Fig. 5B, C). To identify potential epigenetic regulatory sites on the ID3 promoter, DNA methylation method and sequencing was performed on the methylation region of the ID3 gene (Fig. 5D, E). Our data identified several important hypermethylated CpG sites within the ID3 promoter region, including CpG site 11 (−295 position; shLuc methylation = 35.28% vs. shESM1 methylation = 26.45%), CpG site 12 (−285 position; shLuc methylation = 82.06% vs. shESM1 methylation = 61.51%), site CpG site 13 (−275 position; shLuc methylation = 65.04% vs. shESM1 methylation = 49.46%), and CpG site 14 (−266 position; shLuc methylation = 55.43% vs. shESM1 methylation = 48.95%). These results suggested that the methylation of CpG sites (position 11–14) was lower in ESM1 knockdown stable cells than in control cervical cancer cells (Fig. 5F). These results suggest that ESM1 suppresses ID3 expression by promoting methylation of the ID3 promoter in human cervical cancer cells. In addition, to verify whether ID3 expression in cervical cancer is epigenetically inhibited, a methylation inhibitor, 5-AZA, was employed. Western blot analysis showed that 5-AZA treatment significantly upregulated ID3 expression in high ESM1-expressing cervical cancer cell lines (Fig. 5G). Migration and Matrigel invasion assays further showed that 5-AZA treatment significantly inhibited the metastatic abilities of HeLa and SiHa cells (Fig. 5H). Collectively, these data clearly indicate that ESM1 may epigenetically inhibit ID3 expression in cervical cancer.

DNA methylation within CpG islands is primarily catalyzed by DNA methyltransferases (DNMTs) [20]. During this epigenetic regulatory event, de novo methylation is governed by DNMT3A and DNMT3B, while the main function of DNMT1 is to maintain these methylation patterns during cell division [21]. In cervical cancer, the activity and expression of DNMTs are often upregulated and are correlated with advanced disease stages and poor clinical outcomes [6]. To further elucidate whether ESM1 is involved in the epigenetic regulation of ID3 expression, the expression of DNMTs in ESM1 knockdown stable cells was examined using RT-qPCR and immunoblotting. Intriguingly, among DNMT1, DNMT3A, and DNMT3B, ESM1 knockdown upregulated ID3 expression and downregulated only DNMT3A mRNA and protein expression in both cervical cancer cells (Fig. 5I, J). Therefore, the observed upregulation of ID3 in ESM1 knockdown stable cells may result from DNMT3A downregulation and subsequent demethylation of the ID3 promoter.

### ESM1 knockdown suppresses tumorigenic and metastatic potential of cervical cancer cells in vivo

**Fig. 6:**
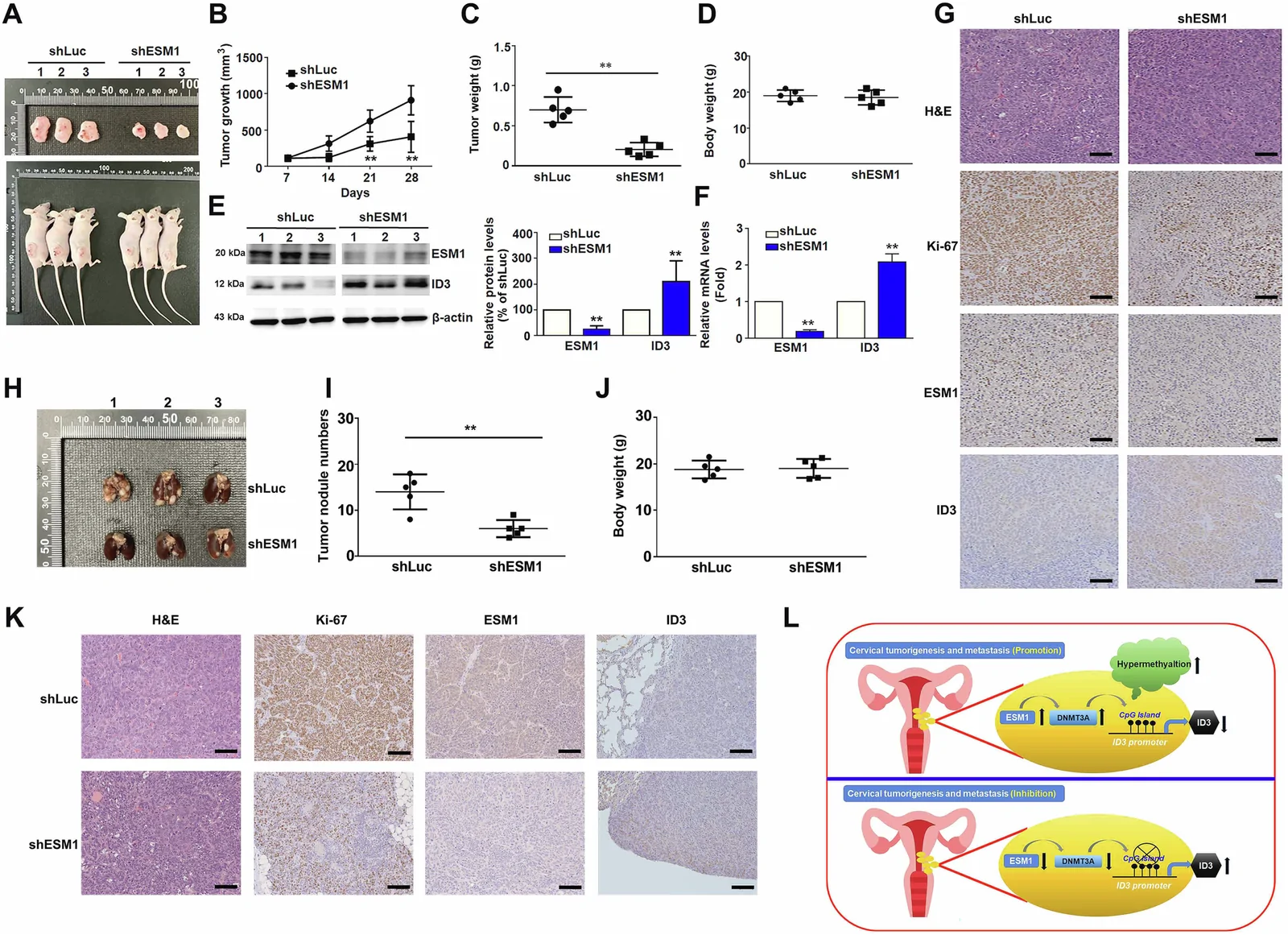
ESM1 knockdown inhibits the tumorigenesis and metastasis of cervical cancer in vivo. The stable control shLuc or shEMS1 cervical cancer cells were subcutaneously inoculated into the flanks of the nude mice to establish xenograft tumors. **A** Representative images of the xenograft tumors and the mice on the day of sacrifice. **B** Tumor size was measured weekly for 4 weeks. **C**, **D** The weights of the tumors and the body weights of tumor- bearing mice on the day of sacrifice. **E** The protein expression of ESM1 and ID3 in tumor tissue was assessed by Western blot. Relative quantification was performed using β-actin as an internal control. **F** The mRNA expression of ESM1 and ID3 in the tumor was examined by RT-qPCR. **G** Representative images of the H&E (hematoxylin and eosin) staining and IHC staining against Ki-67, ESM1, and ID3 of the tumor sections. **H** ESM1 knockdown on cervical cancer metastasis was assessed by injecting the stable control shLuc or shEMS1 cervical cancer cells via the tail vein into C.B17-SCID mice. Representative images of the metastatic nodules of stable cervical cancer cells. **I**, **J** The number of tumor nodules and the body weights of tumor-bearing mice were counted and measured on the day of sacrifice. **K** Representative images of the H&E staining and IHC staining against Ki-67, ESM1, and ID3 of the section of the metastatic tumor nodules. Data are expressed as the mean ± SD of three independent experiments. Fold change relative to the shLuc cells. Scale bar = 50 μm. **L** Image representation of the role of ESM1 and its epigenetic regulation on ID3 through mediating DNMT3A expression in cervical cancer progression.

To investigate the in vivo effect of ESM1 knockdown on cervical cancer cell proliferation, xenograft tumors were established by subcutaneously inoculating ESM1 knockdown stable cells into the flanks of immunodeficient mice. ESM1 knockdown significantly inhibited the size, growth, and weight of xenografted tumors compared to the control tumors (shLuc) (Fig. 6A–C) without affecting the body weight of the host mice (Fig. 6D). To validate our hypothesis, mice were sacrificed, and the harvested xenografted tumors were subjected to immunoblotting, RT-qPCR, and immunohistochemistry (IHC) analysis. Our data demonstrated that ESM1 knockdown significantly upregulated ID3 expression (Fig. 6E, F). The IHC analysis further confirmed this finding, and Ki-67 staining further revealed that ESM1 knockdown also inhibited the proliferation of xenografted cervical cancer cells (Fig. 6G). To further elucidate the in vivo effect of ESM1 knockdown on the metastatic potential of cervical cancer cells, ESM1 knockdown stable cells were inoculated into the C.B17-SCID mice via tail vein injection. After the sacrifice of inoculated mice, we found that knockdown of ESM1 drastically inhibited the formation of metastatic nodules in the lungs (Fig. 6H, I), and the body weight was also not affected by ESM1 knockdown (Fig. 6J). To further evaluate our hypothesis, IHC was used to examine protein expression in lung metastatic nodules from tumor-inoculated mice. As shown in Fig. 6K, knockdown of ESM1 led to upregulation of ID3 and downregulation of Ki-67, a result similar to that observed in the subcutaneous xenograft. These results demonstrated that ESM1 inhibition suppressed the proliferation and metastatic potential while upregulating ID3 expression in vivo. In conclusion, we proposed that in cervical cancer, ESM1 keeps ID3 at low expression by increasing ID3 methylation via DNMT3A, thereby enhancing the malignant properties of cervical cancer (Fig. 6L).

## Discussion

In recent years, accumulating evidence has associated abnormal ESM1 expression with the progression of multiple cancer ranging from glioma to prostate cancer [13]. In cervical cancer, ESM1-mediated tumor progression has been linked to activation of ZEB1/PI3K/Akt and HIF-1α/VEGFα/ERK signaling, leading to enhanced EMT and angiogenesis, respectively [14, 22, 23]. However, the role of ESM1 in the abnormal epigenetic alterations in cervical cancer remains elusive. In this study, we first demonstrated by IHC staining and clinical database analysis that the expression of ESM1 is upregulated in cervical cancer tissue and associated with poor overall survival among cervical cancer patients. Furthermore, in vitro and in vivo experiments both demonstrated that knocking down ESM1 expression significantly inhibits the malignant properties of high-ESM1-expressing cervical cancer cells. The re-emergence of these malignant properties after re-introducing ESM1 expression in stable ESM1 knockdown cervical cancer cells further supports the central role of ESM1 in cervical cancer progression, consistent with previous studies. Surprisingly, the GSEA and clinical database analyses revealed that ID3, a previously unknown ESM1 target, is inversely correlated with ESM1 in cervical cancer, hinting at a missing piece in the current understanding of ESM1-mediated cancer progression.

ID3, a negative transcription regulator, has been shown to be context-dependent in regulating cancer progression. In esophageal cancer [24], and prostate cancer [25], overexpression of ID3 is associated with poor prognosis. In contrast, low ID3 expression in AML (acute myeloid leukemia) [26] and papillary thyroid cancer [18] was associated with poor survival and lymphatic metastasis, respectively. In papillary thyroid cancer, Xu et al. demonstrated that ID3 loss prevents the formation of the ID3/E47 transcription-inhibitory heterodimer, thereby inducing E74-mediated EMT and subsequently increasing the metastatic potential of papillary thyroid cancer cells [18]. Additionally, induction of ID3 expression in squamous carcinoma cells reduced both in vitro and in vivo proliferation by activating Elk1/Caspase-8-dependent apoptosis [27]. In this study, we showed that ectopic expression of ID3 significantly inhibited the malignant properties of high-ESM1-expressing cells, and the inverse correlation between ID3 and ESM1 expression persisted in the in vivo model. Additionally, silencing ID3 expression in stable ESM1 knockdown cervical cancer cells restores the hindered metastatic potential, further implicating the indispensable role of ID3 inhibition in ESM1-mediated cervical cancer progression. Together, these data suggest that ID3 is a bona fide tumor suppressor in cervical cancer.

A growing body of evidence suggests that abnormal epigenetic modifications are another contributor to cervical cancer progression, apart from HPV infection [5]. In addition, previous studies have shown that in both cervical lesions and cervical cancer, the promoter region of tumor suppressor genes is often hypermethylated [28, 29]. Given that ID3 is a tumor suppressor in cervical cancer, we wondered whether it is epigenetically regulated in this disease. UALCAN database analysis showed that the methylation level of the ID3 promoter is higher in cervical cancer tissue, and the elevated promoter methylation is correlated with more advanced grades, stages, and metastasis of cervical cancer. Next-generation methylation sequencing revealed that ESM1 knockdown drastically reduced methylation in the regulatory region (−1 to −1000 bp) of ID3. Additionally, we demonstrated that exposure to 5-AZA, a DNA methyltransferase inhibitor, increased ID3 expression in high-ESM1-expressing cervical cancer cells and inhibited their metastatic potential. Collectively, these data suggest that epigenetic inhibition of ID3 is crucial for the ESM1-mediated cervical cancer progression.

In cervical cancer, the expression and activity of DNMTs are often elevated and associated with poor clinical outcomes [6]. As DNMTs are the key enzymes that govern DNA methylation, DNMT1 primarily maintains de novo methylation initiated by DNMT3A and DNMT3B [21]. Intriguingly, in stable ESM1 knockdown cervical cancer cells, we observed by RT-qPCR that only DNMT3A was downregulated, while DNMT1 and DNMT3B remained unaffected. This suggests that DNMT3A-mediated methylation of the ID3 promoter may be essential to ESM1-mediated cervical cancer progression. On one hand, DNMT3A was found to promote the malignant properties of cervical cancer cells by inhibiting the expression of tumor suppressor genes, including IGFBP3 and TP53AIP, through recruitment of the promoter methylation complex CUL4B/SUV39H1/HP1 or direct promoter binding, respectively [30, 31]. On the other hand, EZH2 has been shown to increase H3K27 histone methylation of DNMT3A, leading to epigenetic downregulation of DNMT3A. Decreased DNMT3A subsequently leads to upregulation of CCL22 and CCR4, which were originally inhibited by DNMT3A-mediated promoter methylation. Finally, the activation of the CCL22/CCR4 axis will lead to the induction of EMT-mediated cervical cancer progression [32]. In addition, increasing evidence suggests that non-coding RNAs are another modulator of DNMT3A expression in cancer [33]. These studies highlighted the complexity of regulation and the subsequent effects on cervical cancer progression of DNMT3A. In gastric cancer, Chien et al. found that ESM1 can activate EGFR/HER3 and the subsequent Akt/STAT3/ANGPT2 pathway, thereby triggering EMT in gastric cancer cells [34]. Interestingly, in pluripotent stem cells, STAT3 can regulate DNMT3A expression in concert with α-ketoglutarate-mediated Otx2 expression, and cancer with STAT3 expression often displays abnormal DNA methylation [35]. This suggests that Akt/STAT3 is a possible mechanism of ESM1-mediated DNMT3A expression.

Our findings, to the best of our knowledge, are the first to depict the tumor-suppressor role of ID3 and to demonstrate the possible mechanism by which ESM1 mediates cervical cancer proliferation and metastatic potential. Knockdown of ESM1 significantly inhibited proliferation, colony formation, and migration/invasion in cervical cancer cells in vitro and in vivo by increasing ID3 expression via inhibition of ID3 promoter DNA methylation. However, the precise mechanisms by which ESM1/DNMT3A regulates epigenetic regulation of ID3 remain elusive. In conclusion, our study provides a novel epigenetic link of ESM1-mediated cervical cancer progression and provides new knowledge for the development of cervical cancer therapy.

## Materials and methods

### Chemical and materials

Fetal bovine serum (FBS) and penicillin/streptomycin were purchased from HyClone (Cytiva, USA). Minimum essential medium (MEM), Dulbecco’s modified Eagle’s medium (DMEM), and Ham’s F-12 nutrient mixture (F12) were obtained from Life Technologies (NY, USA). 3-(4,5-Dimethylthiazol-2-yl)-2,5-diphenyltetrazolium bromide (MTT) and 5-azacytidine (5-AZA) were purchased from Sigma-Aldrich, Inc (Louis, USA). The transfection reagents Lipofectamine 3000 and RNAiMAX were obtained from Thermo Fisher Scientific, Inc. (Waltham, MA, USA). PCR primers were synthesized by Mission Biotech (Taipei, Taiwan); shRNA-Luc, shRNA-ESM1 vectors, and the plasmids required for lentiviral packaging were purchased from the RNA Technology Platform and Gene Manipulation Core facility of Academia Sinica (Taipei, Taiwan). Empty vectors, ESM1 expression vectors, and ID3 expression vectors were obtained from GENEWIZ Company (Takeley, UK). siRNA-control and siRNA-ID3 were synthesized by Santa Cruz Biotechnology (Dallas, USA). A human cervical cancer array (CR804 and CR501) was purchased from US Biomax, Inc, including human normal cervical tissue (CR804; *N* = 10) and cervical squamous cell carcinoma tissues (CR501; *N* = 40).

### Cell culture

The human cervical cancer cell lines HeLa and C33-A were obtained from the Bioresource Collection and Research Center (BCRC) of the Food Industry Research and Development Institute (FIRDI) (Hsinchu, Taiwan) and maintained in DMEM/F12 and MEM, respectively. SiHa cells were purchased from the American Type Culture Collection (ATCC) (Rockville, MD, USA) and maintained in DMEM. The growth medium of the human cervical cancer cell lines was formulated by supplementation with 10% FBS and 1% penicillin/streptomycin. Human cervical epithelial cells (HCerEpiC) were obtained from ScienCell Research Laboratories (Carlsbad, CA, USA) and maintained in CerEpiCM (Cervical Epithelial Cell Medium) supplemented with CerEpiCGS (CerEpiC growth supplement) according to the manufacturer’s instructions. All the cells were maintained in a humidified incubator at 37 °C and 5% CO_2_.

### Cell growth assay

Cervical cancer cells (4 × 10^4^/well) were seeded in 24-well plates and cultured in growth medium for the indicated times. The medium was then replaced with MTT solution (0.5 mg/mL in growth medium), and the cells were incubated for an additional 2 h. Cell growth was determined by measuring the absorbance (570 nm) of the MTT reduction products produced by mitochondrial dehydrogenases dissolved in methanol. The area under the curve (AUC) from 24 to 96 h was measured using the trapezoidal method in GraphPad Prism software.

### Colony formation assay

Cervical cancer cells (5 × 10^3^/well) were seeded in 6-well plates and incubated for 14 days. Crystal violet was used to visualize the colonies formed. The number of surviving colonies was counted under a microscope, and the control group was used to normalize the relative colony formation index.

### Cell migration and Matrigel invasion assay

A 48-well Boyden chamber containing a polycarbonate (PC) membrane with 8 µm pores was used to evaluate the migration and invasion of cervical cancer cells with or without Matrigel coating prior to cell seeding. After loading the lower chamber with growth medium, the cervical cancer cells were then seeded into the upper chamber with serum-free medium. The assembled chambers were then incubated for 16 and 24 h for the migration and invasion assays, respectively. Giemsa staining was used to visualize the migrated cells, and the number of migrated cells was counted in at least 5 random fields under a microscope.

### Western blot analysis

Total cell lysate was obtained by incubating the indicated cells in NETN buffer, and the protein concentration was then quantified using Bradford assay (Thermo Fisher Scientific). Equal amounts of denatured total protein (20 µg) were separated by SDS‒PAGE and transferred onto a PVDF membrane. The protein-containing PVDF membrane was incubated with blocking buffer to prevent nonspecific antibody binding. To detect changes in protein expression, the blocked PVDF membrane was incubated overnight at 4 °C with primary antibodies against ESM1 (#AF1810; R&D Systems; 1:1000), ID3 (A5375; BioAb, Inc; 1:1000), DNMT3A (#3598; Cell Signaling Technology, Inc; 1:1000), and β-actin (sc-69879; Santa Cruz Biotechnology; 1:5000). After the primary antibody-bound PVDF membranes were incubated with an HRP-conjugated secondary antibody (1:10000), the proteins were visualized by incubating the membranes with ECL reagents to generate a chemiluminescent signal. β-actin was used as an internal control.

### qRT-PCR assay

Total cell RNA was isolated using TRIzol reagent as described previously. The extracted RNA was quantified, and 1 µg of total RNA was used for the synthesis of cDNA with the GoScript reverse transcription system according to the manufacturer’s instructions. Quantitative amplification of the synthesized cDNA was performed using GoTaq qPCR Master Mix and specific PCR primers targeting ESM1: (F) 5′-AGCAATAATTATGCGGTGGACT-3′, (R) 5′-ACCAAACTCTTCACCAAAAGGA-3′; ID3: (F) 5′-GAGCTTTTGCCACTGACTCG-3′, (R) 5′-GTGAAGTCAAGTGGGCAGGG-3′; DNMT1: (F) 5′-ACCTACCACGCCGACAT-3′, (R) 5′-AGGTCCTCTCCGTACTCCA-3′; DNMT3A: (F) 5′-TTCTACCGCCTCCTGCATGAT-3′, (R) 5′-GCGAGATGT CCCTCTTGTCACTA-3′, DNMT3B: (F) 5′-AGGGAAGACTCGATCCTCGTC-3′, (R) 5′-GTGTGTAGCTTAGCAGACTGG-3′ and GAPDH: (F) 5′-CATCATCCCTGCCTC TACTG-3′, (R) 5′-GCCTGCTTCACCACCTTC-3′ (Mission Biotech, Taipei, Taiwan). The 2^-ΔΔCT^ method was used to determine the relative expression of each mRNA, with GAPDH used as an internal control.

### Overexpression-ID3/ESM1 or Knockdown-ID3/ESM1 assay

Cervical cancer cells were seeded into cell culture dishes at concentrations that achieved 70% confluency on the day of transfection. After the Ov-ID3 plasmid (1 µg) or siRNA (100 nM) was mixed with Lipofectamine 3000 or Lipofectamine RNAiMAX in Opti-MEM, the mixture was added to the growth medium and incubated for 48 h to establish transient transfection. To further establish stable ESM1 knockdown cervical cancer cell lines, lentiviruses containing the puromycin resistance gene and pLKO.1 shRNA targeting luciferase (scramble control) or ESM1 were produced using HEK293T cells. After the supernatants containing lentiviruses were collected and filtered (0.45 µm), the cervical cancer cells were subjected to polybrene (8 µg/mL)-assisted infection with either shLuc or shESM1 lentivirus. Puromycin (2 µg/mL) was used to select the successfully transduced cervical cancer cells.

### RNA sequences

Total RNA was extracted from shLuc and shESM1 cells by using TRIzol reagent. The RNA sequence were analysis by BIOTOOLS company (New Taipei, Taiwan). RNA integrity was assessed using an Agilent 2100 Bioanalyzer (Agilent Technologies, USA). Library quality was assessed using paired-end sequencing was performed on the Illumina NovaSeq 6000 platform (Illumina, USA). Differential expression analysis was performed using the DESeq2 method, and genes with an adjusted *p* value < 0.05 and a fold change > 1.0 between the two groups were considered differentially expressed genes (DEGs). Integrated database and functional analyses, including principal component analysis (PCA), Kyoto Encyclopedia of Genes and Genomes (KEGG) and enrichment analysis were performed with RNAseq Cloud Platform. The RNA sequencing data have been uploaded and are publicly accessible in the RNA-seq database repository. (DOI: 10.5281/zenodo.20272590).

### DNA methylation assay

Sodium bisulfite treatment of DNA was used to convert unmethylated cytosine (C) into uracil (U), after which the DNA was purified by QIAGEN EpiTect® Bisulfite Kits. Genome-wide methylation analysis was conducted by Mission Biotech Co., Ltd. (Taipei, Taiwan).

### Xenograft mouse model and tail-vein tumor metastasis assay

Female nude mice (5-week-old BALB/c) were obtained from the National Laboratory Animal Center (Taipei, Taiwan). The stable control shLuc or shEMS1 cervical cancer cells were subcutaneously inoculated into the flanks of the nude mice to establish xenograft tumors (*n* = 5 for each group). Weekly measurements of tumor size and body weight were carried out for 4 weeks. Mice were euthanized when tumors reached 2000 mm³ in volume or upon the occurrence of tumor ulceration/bleeding. After the final measurement (28 days), the mice were sacrificed, and the xenograft tumors were harvested for Western blotting, qRT‒PCR, and immunohistochemistry analysis. To further assess the in vivo metastasis, stable control shLuc or shEMS1 cervical cancer cells were introduced into 5-week-old female C.B17-SCID mice via tail vein injection (*n* = 5 for each group). After eight weeks, the mice were sacrificed, and body weights were recorded before lung tissue collection. Collected tumor-bearing lung tissues were subjected to IHC analysis to determine the expression of Ki-67, ESM1, and ID3.

### Bioinformatic analysis of clinical cervical cancer database

The expression levels of ESM1 and ID3 in normal tissues and cervical cancer patients were analysed using the UALCAN database (https://ualcan.path.uab.edu/index.html), TNMplot V2 analytical platform (https://tnmplot.com/analysis/) and GEO database (GSE9750 and GSE6791). The promoter methylation status of ID3 in normal and cervical cancer patients, stratified by tumor grade, tumor stage, and nodal metastasis status (N0/N1), was evaluated using UALCAN (https://ualcan.path.uab.edu/index.html). The overall survival (OS) of cervical cancer patients was assessed using the Kaplan–Meier plotter database (https://kmplot.com/analysis/). The correlation between ESM1 and ID3 expression was determined using the TNMplot V2 database (https://tnmplot.com/analysis/).

### Immunohistochemistry (IHC)

The shLuc or shESM-bearing lung tissues sections were deparaffinized and rehydrated by using VECTASTAIN Universal Elite ABC Kit (Vector Labs). Antigen retrieval was conducted by heating the sections in High pH Antigen Unmasking Solution (Vector Laboratories, USA). These tissues sections were then incubated overnight at 4 °C with primary antibodies against Ki-67 (ab16667, Abcam, 1:200), ESM1 (#AF1810; R&D Systems, 1:1000), ID3 (A5375; BioAb, Inc, 1:1000) and added the biotinylated secondary antibodies and subsequently treated with the VECTASTAIN Universal Elite ABC Kit (Vector Laboratories, USA). The sections were counterstained with hematoxylin and photographed under a light microscope.

### Statistical analysis

All the statistical analyses were performed on GraphPad Prism 6 (San Diego, CA, USA). The statistical significance of differences between single- or multiple-group comparisons was evaluated using Student’s *t* test or one-way ANOVA (with Dunnett’s correction), respectively. Data are expressed as the mean ± SD of three biological repetitions. Differences were considered significant if the *p* value was less than 0.05 or 0.01.

## Supplementary information

Supplementary Figure S1

Original-data

## Data Availability

The data analyzed during this study are included in this manuscript.
