# Supplementary material for: ESM1-mediated DNMT3A suppresses cervical cancer metastasis through epigenetic regulation of ID3 expression
Source: Cell Death Discov. 2026 Aug 7;12:438. doi: 10.1038/s41420-026-03239-z (PMC13736847; doi:10.1038/s41420-026-03239-z)
Supplement: Supplementary file 1 [file 41420_2026_3239_MOESM2_ESM.pdf]

Fig.1F

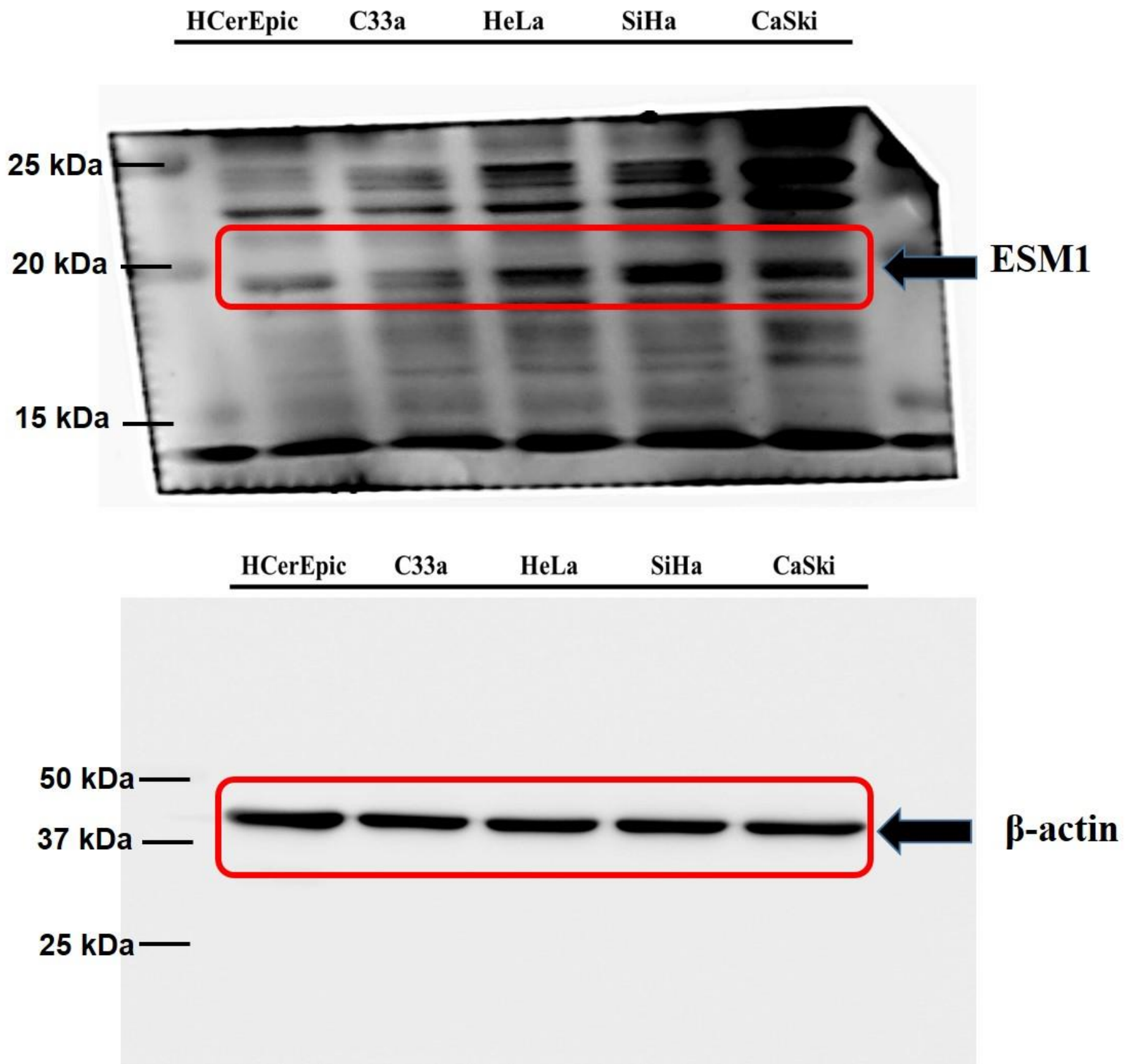
HCerEpic C33a HeLa SiHa CaSki
25 kDa
20 kDa
15 kDa
ESM1
HCerEpic C33a HeLa SiHa CaSki
50 kDa
37 kDa
25 kDa
β-actin

Fig.2A

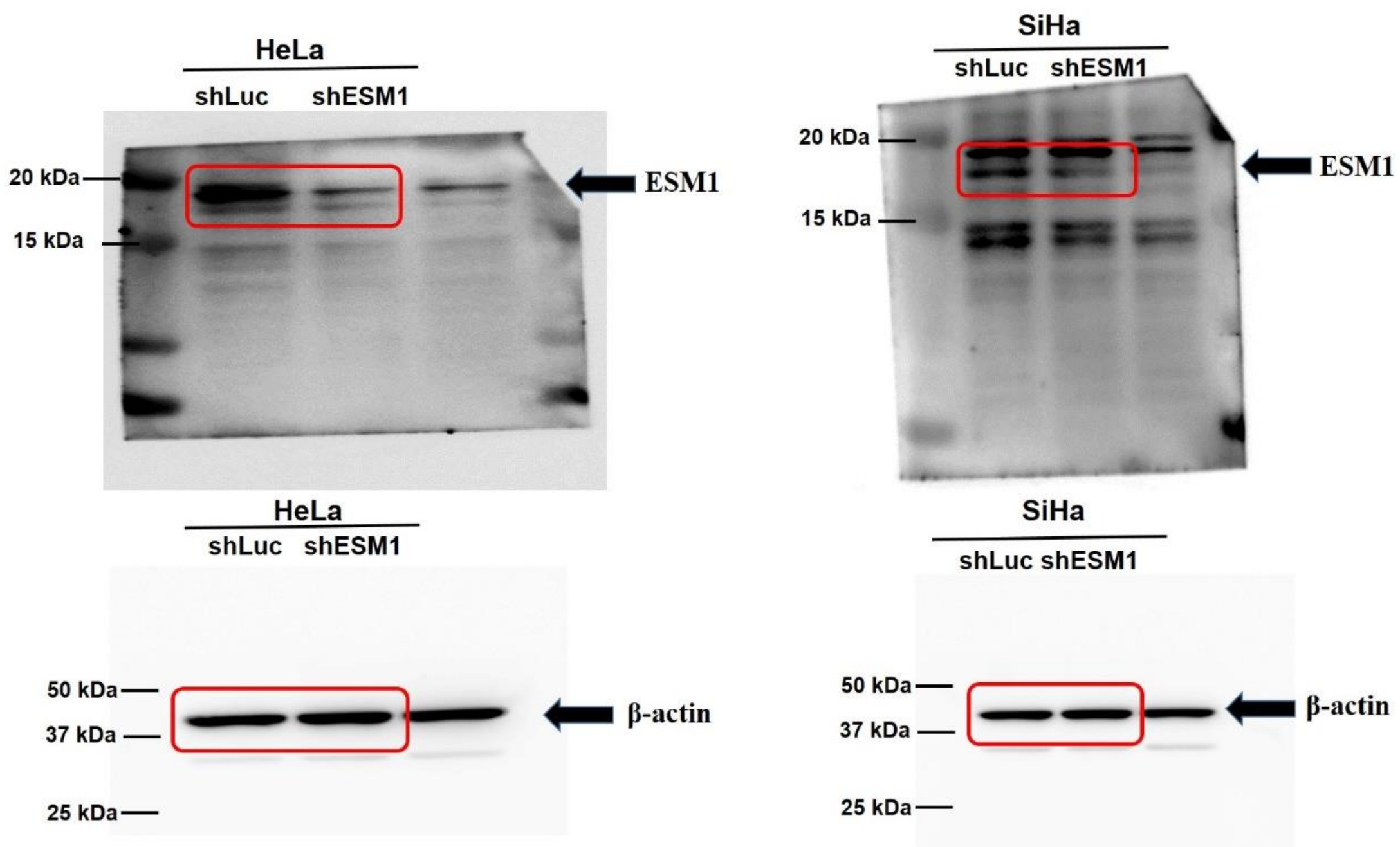
HeLa
shLuc shESM1
20 kDa
15 kDa
ESM1
SiHa
shLuc shESM1
20 kDa
15 kDa
ESM1
HeLa
shLuc shESM1
50 kDa
37 kDa
25 kDa
β-actin
SiHa
shLuc shESM1
50 kDa
37 kDa
25 kDa
β-actin

Fig.3C

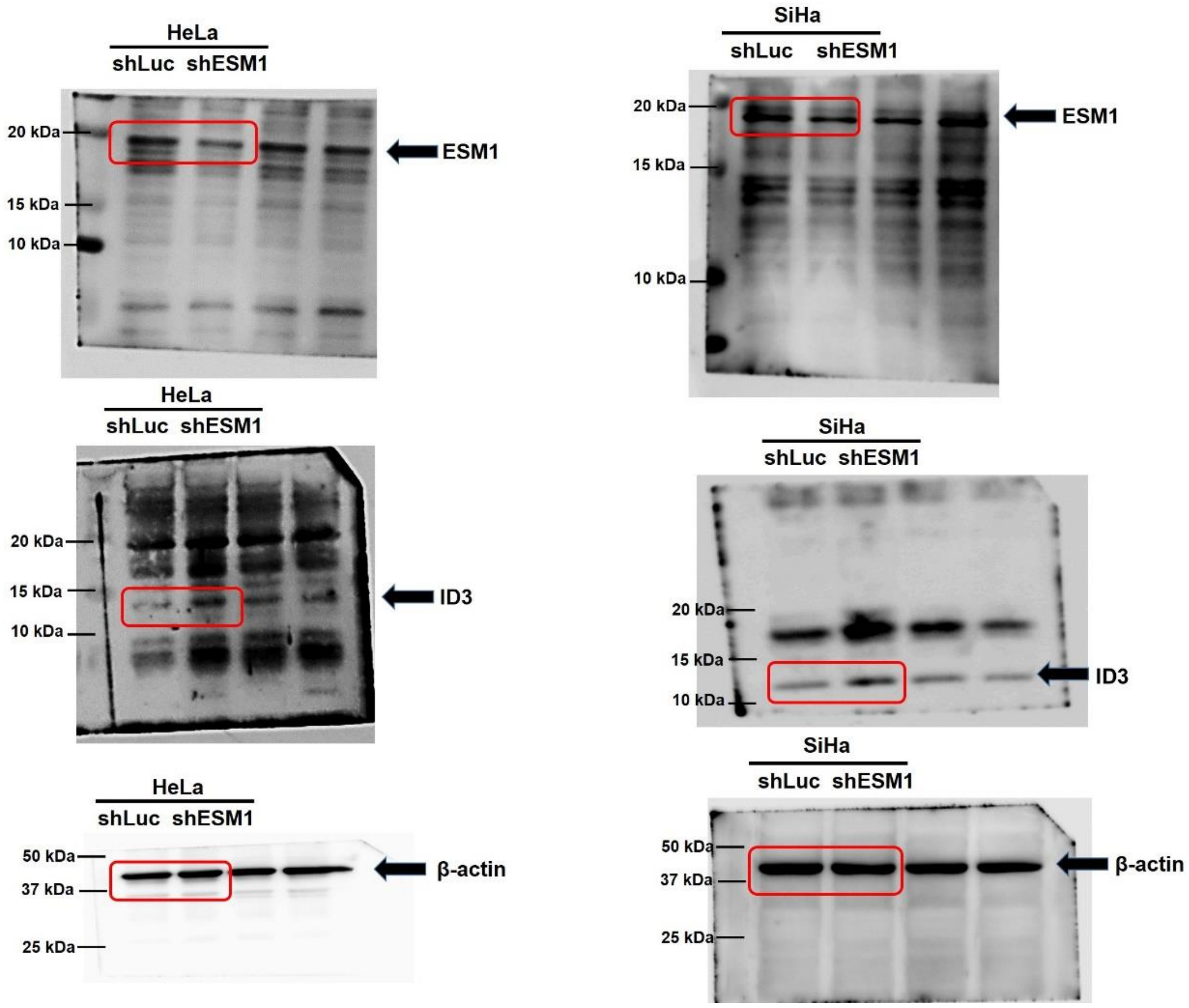
HeLa
shLuc shESM1
20 kDa
15 kDa
10 kDa
ESM1
SiHa
shLuc shESM1
20 kDa
15 kDa
10 kDa
ESM1
HeLa
shLuc shESM1
20 kDa
15 kDa
10 kDa
ID3
SiHa
shLuc shESM1
20 kDa
15 kDa
10 kDa
ID3
HeLa
shLuc shESM1
50 kDa
37 kDa
25 kDa
β-actin
SiHa
shLuc shESM1
50 kDa
37 kDa
25 kDa
β-actin

Fig.3H

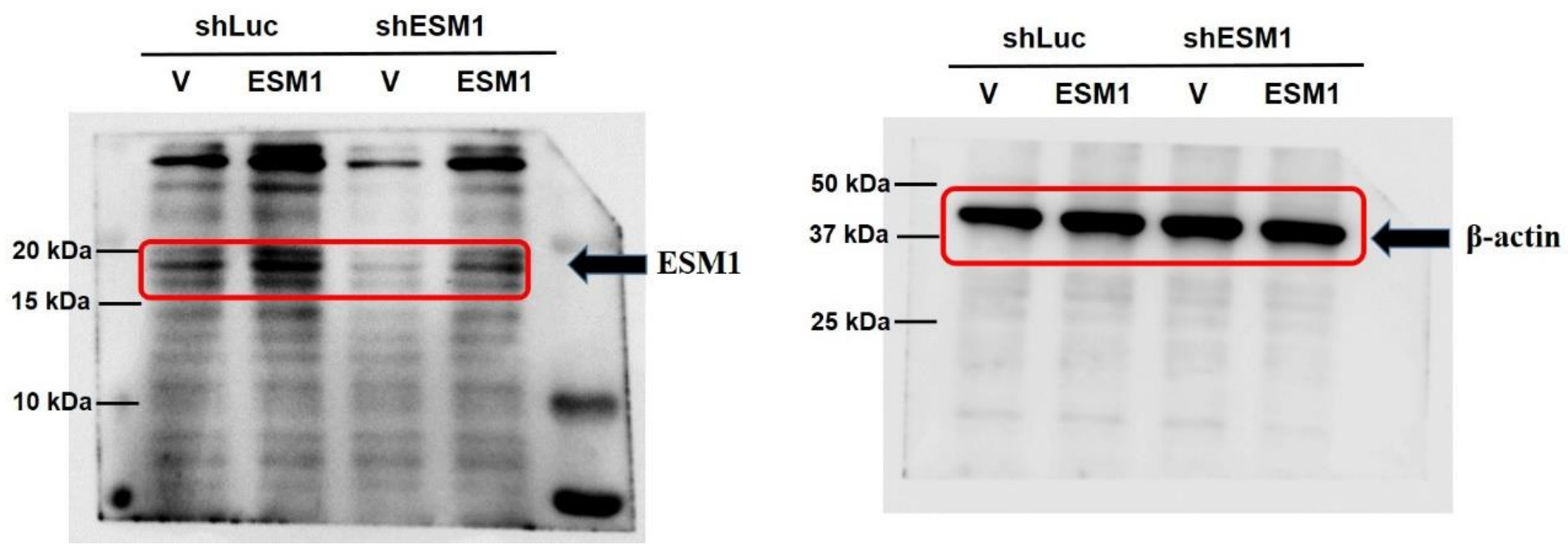
shLuc shESM1
V ESM1 V ESM1
20 kDa
15 kDa
10 kDa
ESM1
shLuc shESM1
V ESM1 V ESM1
50 kDa
37 kDa
25 kDa
β-actin

Fig.4A

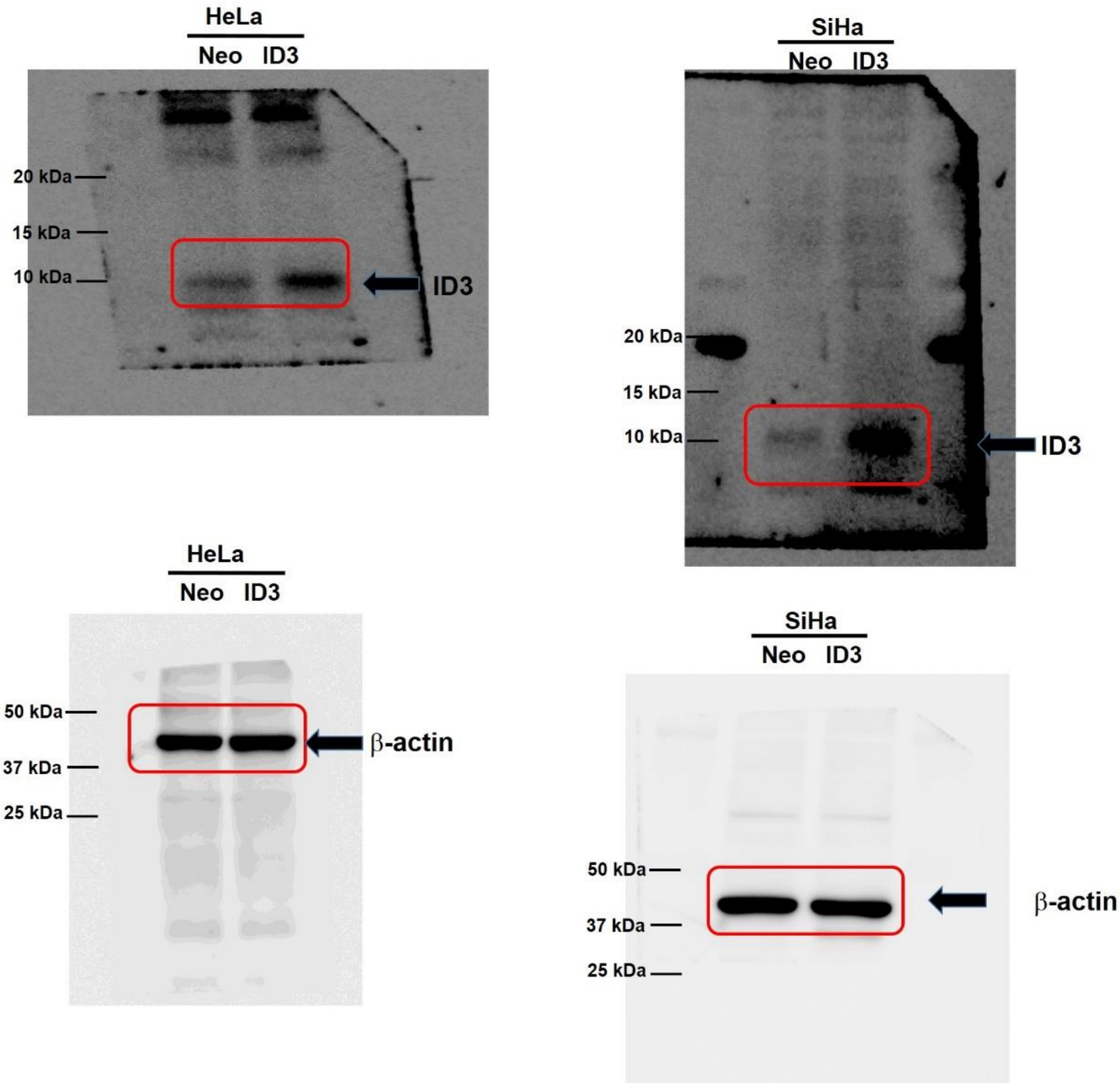
HeLa
Neo ID3
20 kDa
15 kDa
10 kDa
ID3
SiHa
Neo ID3
20 kDa
15 kDa
10 kDa
ID3
HeLa
Neo ID3
50 kDa
37 kDa
25 kDa
β-actin
SiHa
Neo ID3
50 kDa
37 kDa
25 kDa
β-actin

Fig.4E.4F

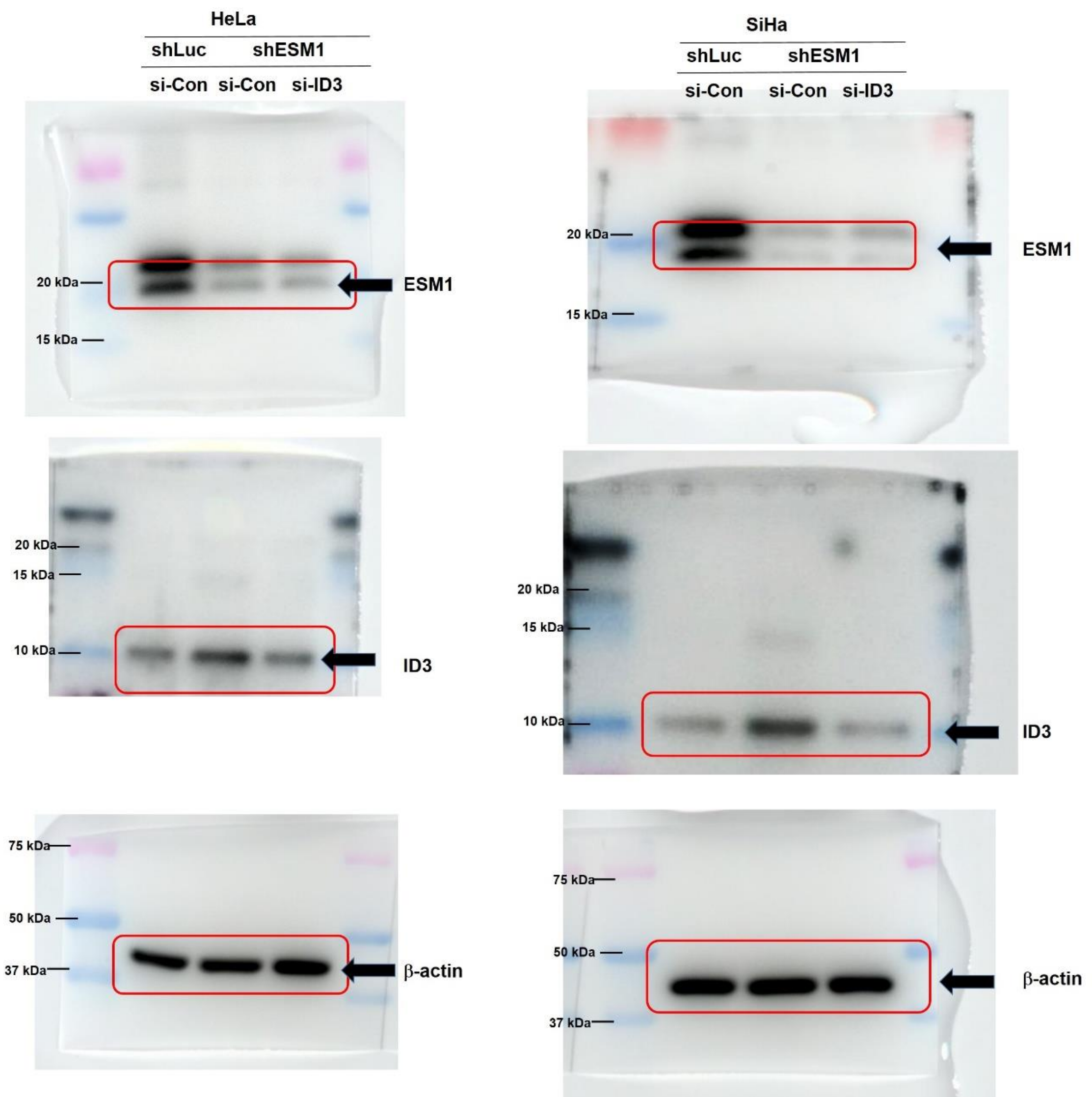

HeLa
shLuc
shESM1
si-Con
si-Con
si-ID3
20 kDa
15 kDa
ESM1
20 kDa
15 kDa
10 kDa
ID3
75 kDa
50 kDa
37 kDa
β-actin
SiHa
shLuc
shESM1
si-Con
si-Con
si-ID3
20 kDa
15 kDa
ESM1
20 kDa
15 kDa
10 kDa
ID3
75 kDa
50 kDa
37 kDa
β-actin

Fig.5G

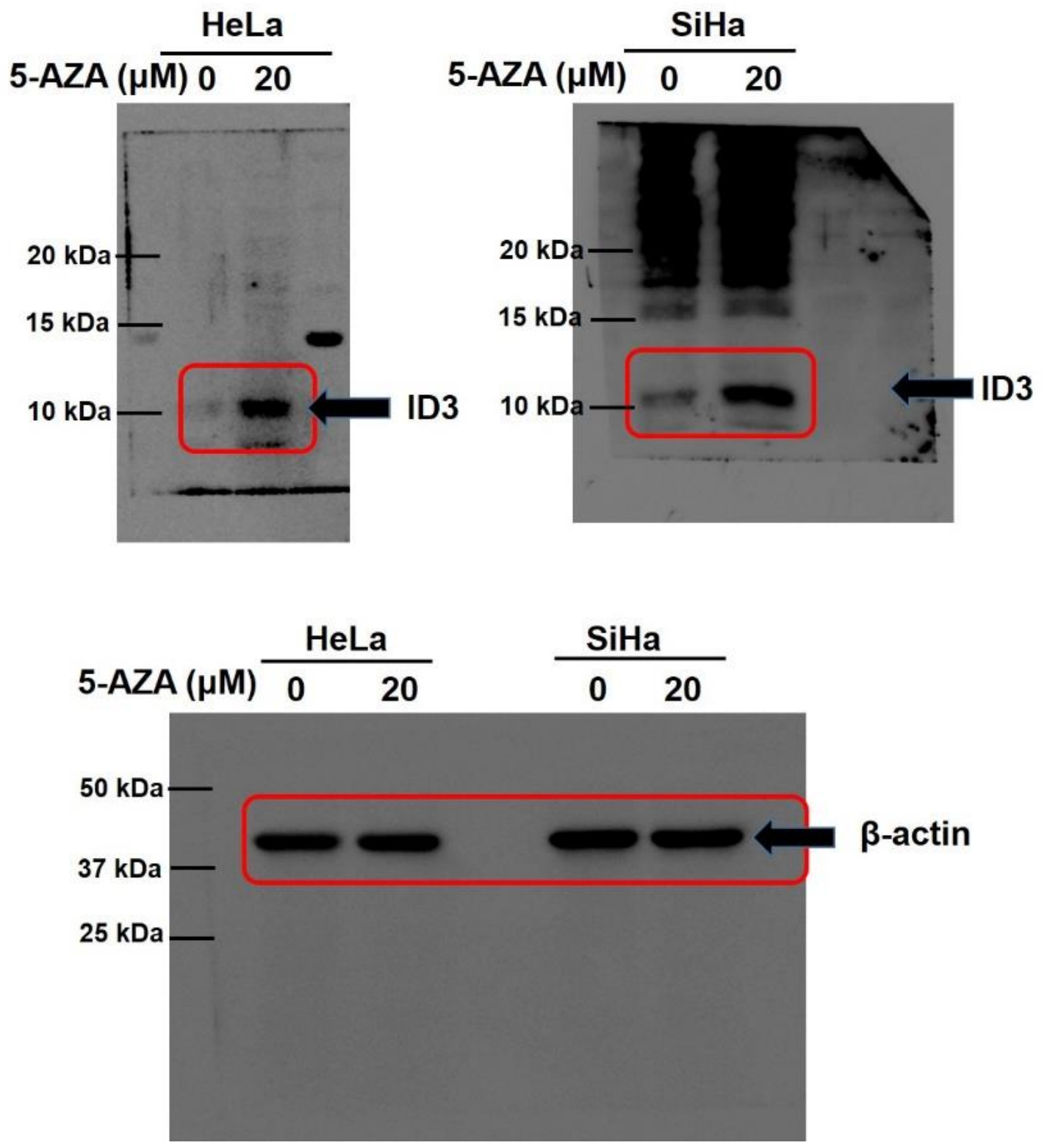
HeLa
5-AZA (μM) 0 20
20 kDa
15 kDa
10 kDa
ID3
SiHa
5-AZA (μM) 0 20
20 kDa
15 kDa
10 kDa
ID3
HeLa
SiHa
5-AZA (μM) 0 20 0 20
50 kDa
37 kDa
25 kDa
β-actin

Fig.5J

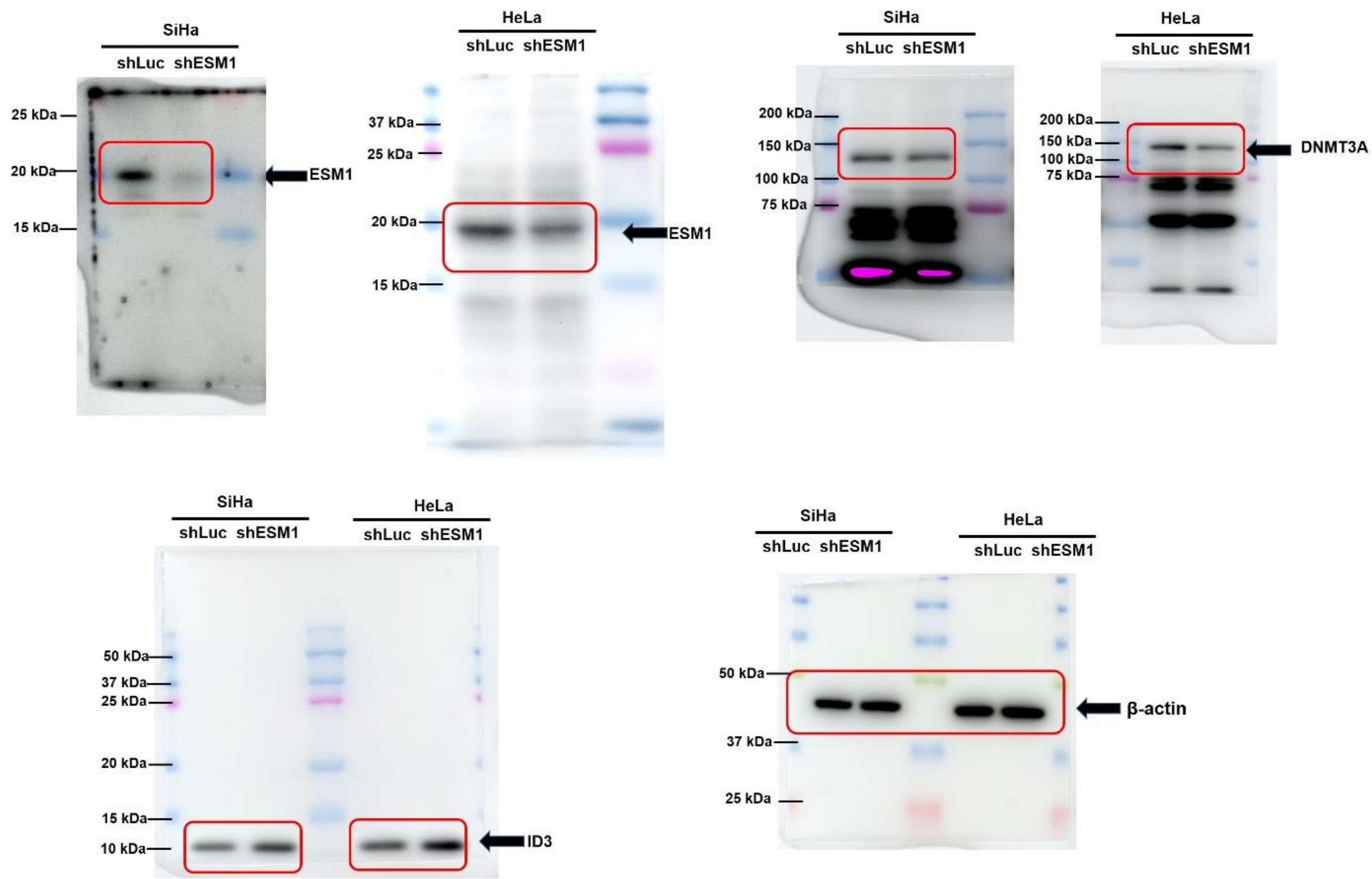
SiHa
shLuc shESM1
25 kDa
20 kDa
15 kDa
ESM1
HeLa
shLuc shESM1
37 kDa
25 kDa
20 kDa
15 kDa
ESM1
SiHa
shLuc shESM1
200 kDa
150 kDa
100 kDa
75 kDa
HeLa
shLuc shESM1
200 kDa
150 kDa
100 kDa
75 kDa
DNMT3A
SiHa
shLuc shESM1
HeLa
shLuc shESM1
50 kDa
37 kDa
25 kDa
20 kDa
15 kDa
10 kDa
ID3
SiHa
shLuc shESM1
HeLa
shLuc shESM1
50 kDa
37 kDa
25 kDa
β-actin

Fig.6E

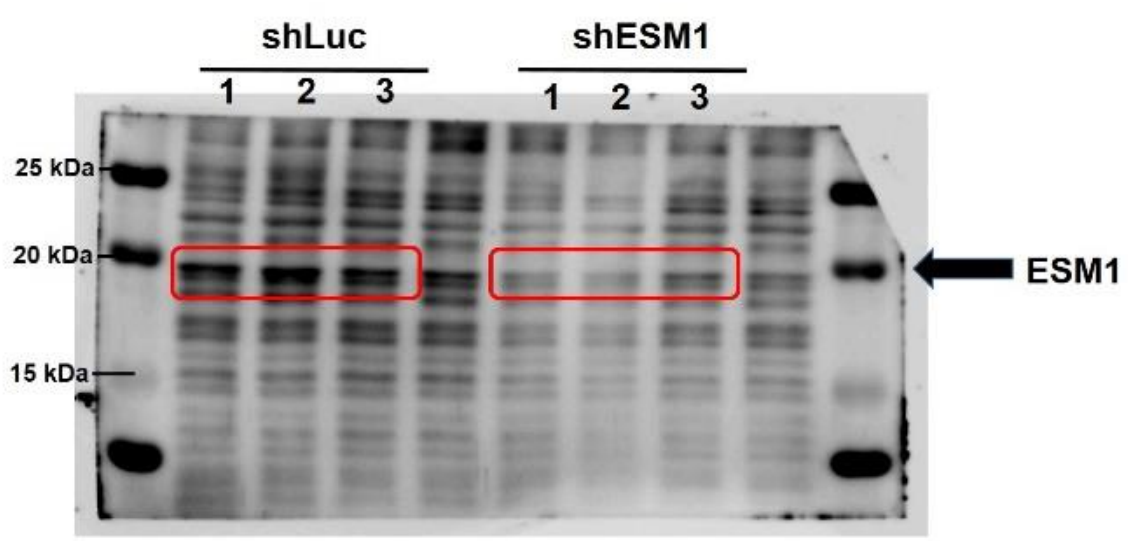

shLuc
shESM1
1 2 3
1 2 3
25 kDa
20 kDa
15 kDa
ESM1

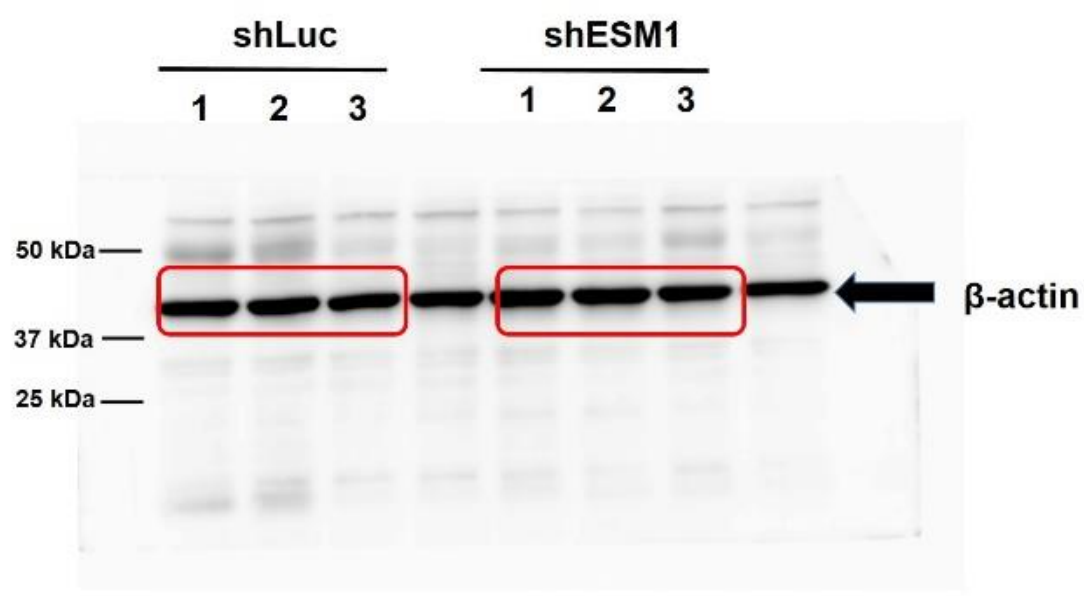

shLuc
shESM1
1 2 3
1 2 3
50 kDa
37 kDa
25 kDa
β-actin

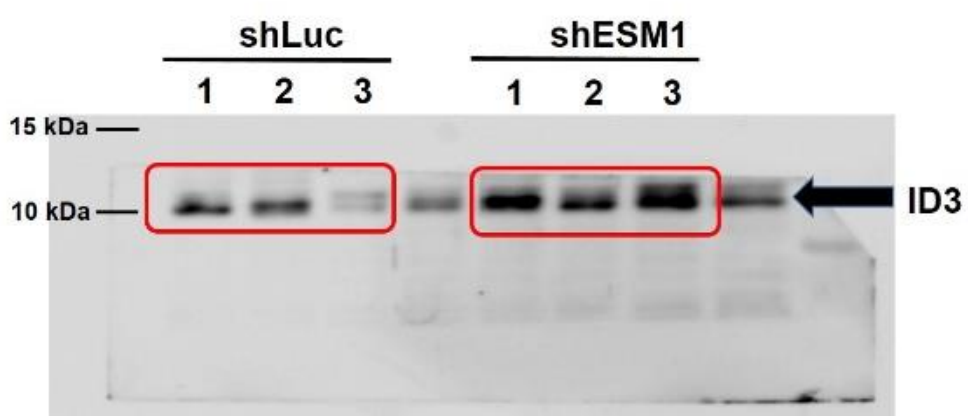

shLuc
shESM1
1 2 3
1 2 3
15 kDa
10 kDa
ID3
