# Supplementary material for: ESM1-mediated DNMT3A suppresses cervical cancer metastasis through epigenetic regulation of ID3 expression
Source: Cell Death Discov. 2026 Aug 7;12:438. doi: 10.1038/s41420-026-03239-z (PMC13736847; doi:10.1038/s41420-026-03239-z)
Supplement: Supplementary file 2 [file 41420_2026_3239_MOESM1_ESM.docx]

**ESM1-mediated DNMT3A suppresses cervical cancer metastasis through epigenetic regulation of ID3 expression**

Chen-Lin Yu, Chia-Liang Lin, Hsiang-Lin Lee, Hui-Ling Chiou, Pei-Ni Chen, Shun-Fa Yang, Yi-Hsien Hsieh

**Supplementary Figure S1**

**Supplementary Figure S1**. ESM1 regulates the malignant properties of human cervical cancer HeLa cells (A) The reintroduction of ESM1 expression by the ESM1-expressing plasmid was confirmed by Western blot. (B) Cell growth was assessed by MTT assay. (C) Cell proliferation was evaluated by colony formation assay. (D) Cell migration and invasion were examined using migration and Matrigel invasion assays. Data are expressed as the mean ± SD of three independent experiments. Each experiment values are relative to the shLuc group. ** p < 0.01 compared with the shLuc control group. # p < 0.05 compared with the stable ESM1 knockdown cells transfected with empty vectors.
